# Occurrence and Characteristics of ESBL- and Carbapenemase- Producing *Escherichia coli* from Wild and Feral Birds in Greece

**DOI:** 10.3390/microorganisms10061217

**Published:** 2022-06-14

**Authors:** Zoi Athanasakopoulou, Celia Diezel, Sascha D. Braun, Marina Sofia, Alexios Giannakopoulos, Stefan Monecke, Dominik Gary, Domenique Krähmer, Dimitris C. Chatzopoulos, Antonia Touloudi, Periklis Birtsas, Matina Palli, Giorgos Georgakopoulos, Vassiliki Spyrou, Efthymia Petinaki, Ralf Ehricht, Charalambos Billinis

**Affiliations:** 1 Faculty of Veterinary Science, University of Thessaly, 43100 Karditsa, Greece; zathanas@uth.gr (Z.A.); msofia@uth.gr (M.S.); algiannak@uth.gr (A.G.); atoul@uth.gr (A.T.); 2 Leibniz Institute of Photonic Technology (IPHT), 07745 Jena, Germany; celia.diezel@leibniz-ipht.de (C.D.); sascha.braun@leibniz-ipht.de (S.D.B.); stefan.monecke@leibniz-ipht.de (S.M.); ralf.ehricht@leibniz-ipht.de (R.E.); 3 InfectoGnostics Research Campus, 07745 Jena, Germany; 4 fzmb GmbH, Forschungszentrum für Medizintechnik und Biotechnologie, 99947 Bad Langensalza, Germany; dgary@fzmb.de (D.G.); dkraehmer@fzmb.de (D.K.); 5 Faculty of Public and One Health, University of Thessaly, 43100 Karditsa, Greece; dchatzopoulos@uth.gr; 6 Faculty of Forestry, Wood Science and Design, 43100 Karditsa, Greece; birtsas@uth.gr; 7 Wildlife Protection & Rehabilitation Center, 24400 Gargalianoi, Greece; keppazzgr@gmail.com (M.P.); keppazgr@gmail.com (G.G.); 8 Faculty of Animal Science, University of Thessaly, 41110 Larissa, Greece; vasilikispyrou@uth.gr; 9 Faculty of Medicine, University of Thessaly, 41500 Larissa, Greece; petinaki@uth.gr; 10 Institute of Physical Chemistry, Friedrich-Schiller-University, 07745 Jena, Germany

**Keywords:** ESBL, carbapenemases, *bla*_CTX-M_, *bla*_TEM_, bla_NDM_, *Escherichia coli*, multidrug-resistance, antimicrobial resistance genes, wild birds, Greece

## Abstract

Wild and feral birds are known to be involved in the maintenance and dissemination of clinically-important antimicrobial-resistant pathogens, such as extended-spectrum β-lactamase (ESBL) and carbapenemase-producing Enterobacteriaceae. The aim of our study was to evaluate the presence of ESBL- and carbapenemase-producing *Escherichia coli* among wild and feral birds from Greece and to describe their antimicrobial resistance characteristics. In this context, fecal samples of 362 birds were collected and cultured. Subsequently, the antimicrobial resistance pheno- and geno-type of all the obtained *E. coli* isolates were determined. A total of 12 multidrug-resistant (MDR), ESBL-producing *E. coli* were recovered from eight different wild bird species. Eleven of these isolates carried a *bla*_CTX-M-1_ group gene alone or in combination with *bla*_TEM_ and one carried only *bla*_TEM_. AmpC, fluoroquinolone, trimethoprim/sulfamethoxazole, aminoglycoside and macrolide resistance genes were also detected. Additionally, one carbapenemase-producing *E. coli* was identified, harboring *bla*_NDM_ along with a combination of additional resistance genes. This report describes the occurrence of ESBL- and carbapenemase-producing *E. coli* among wild avian species in Greece, emphasizing the importance of incorporating wild birds in the assessment of AMR circulation in non-clinical settings.

## 1. Introduction

Antimicrobial resistance (AMR) has recently been declared by the World Health Organization as one of the ten most important public health threats faced by humanity [1]. Among Gram-negative bacteria, the major driving force of resistance is the occurrence of β-lactamases, a family of enzymes capable of hydrolyzing the amide bond of the β-lactam ring and, therefore, of rendering β-lactam antimicrobials ineffective [2]. The global establishment and spread of extended-spectrum β-lactamase-producing Enterobacteriaceae (ESBL-PE) and of carbapenemase-producing Enterobacteriaceae (CPE) can be described as one of the most devastating pandemics of multidrug-resistant (MDR) organisms to date. Historically, MDR bacteria have affected patients in hospital settings, where factors such as exposure to antibiotics, artificial ventilation or catheterization provide an enhanced risk for acquisition [3,4]. However, over the last decades, both ESBL-PE and CPE are frequently detected in the community, as well as among domesticated and wild animals and in the environment [5,6]. The localization of ESBL- and carbapenemase-encoding genes on mobile genetic elements (MGEs) that can be efficiently disseminated between different bacteria and hosts, has rendered ESBL-PE and CPE a One Health problem. For instance, integrons associated with plasmids contribute to the spread of AMR due to their mobility, ability to capture different resistance genes and capacity to cluster resistance genes into complex operons, which can be expressed together and jointly diffused by horizontal gene transfer [7]. In addition to antibiotic resistance genes, ESBL and carbapenemase plasmids commonly also harbor non-resistance factors, including toxin–antitoxin systems that are related to the stable inheritance of the respective resistance genes, even in the absence of selective pressure [8].

During the past few years, antibiotic-resistant bacteria from wildlife have received increasing interest from the scientific community, and the potential contribution of wild fauna as an AMR contamination source has been widely acknowledged [9]. Special attention has been given to the role of wild and feral birds, due to their wide exposure to anthropogenic environments, relative abundance and long-range movements. Subsequently, wild and feral avian species are deemed as a reservoir and vehicle for AMR dissemination [10]. Indeed, several previous studies have identified clinically relevant resistance determinants, such as the ESBL gene *bla*_CTX-M-15_ and the carbapenemase gene *bla*_KPC_, among Enterobacteriaceae isolated from wild and feral birds [11,12,13]. Of note, some of these isolates additionally belonged to sequence types frequently detected in human and animal clinical cases, underlining their possible interspecies transmission [14].

Regarding bacterial species, β-lactamase research has been mainly focused on *E. coli*, given its importance as a human and animal pathogen, its commensal nature and the fact that it can be easily disseminated in different ecosystems, enabling the direct comparison of resistance phenotypes in distinct hosts [9,15]. Enzymes of the CTX-M family, particularly those of group 1, are the most common ESBLs among *E. coli* of human, livestock and wild animal origin worldwide [10]. Concerning carbapenemases, Ambler class A variants of the KPC family have the most extensive global distribution among human isolates, followed by class B metallo-β lactamases (MBLs), which are mostly prevalent in Asia, and class D OXA-type genes that are commonly found in Mediterranean countries [16,17,18]. Correspondingly, the carbapenemase genes identified among animal strains in different countries reflect the types of carbapenemases that prevail in human isolates within these regions [15].

Greece, due to its geographic location within the eastern Mediterranean on the intersection of three continents, constitutes an important habitat for both sedentary and migratory wild birds. High ESBL-PE prevalence and endemicity of CPE among humans has been reported [19,20,21,22,23,24,25], however, data regarding the presence of ESBL-PE and CPE among wild birds remain scarce and no study detailing their prevalence and characteristics is available to date. In this context, the objective of the present study was to determine the occurrence and the molecular traits of ESBL- and carbapenemase-producing *E. coli* among wild and feral birds from Greece.

## 2. Materials and Methods

### 2.1. Sample Collection

Between 2019 and 2021, a total number of 362 non-duplicated fecal samples were collected from 47 different wild and feral bird species (Table 1), originating from 23 regional units of Greece. Samples were obtained by inserting a sterile cotton swab into recently deposited feces or directly into the cloaca of live, captured wild and feral birds. Birds were captured using Larsen-type traps, Australian-type traps or modified bird-catching nets, and immediately released after sampling, according to Greek legislation. Samples were only collected following bird species identification. The swabs were transported in Amies medium (Transwab^®^ Amies, Corsham, UK) under refrigeration and arrived in the laboratory within 48 h of their collection.

### 2.2. Isolation, Identification and Antimicrobial-Resistance Phenotype of ESBL- and Carbapenemase-Producing Enterobacteriaceae

Swabs were directly streaked onto both ESBL selective media (CHROMID^®^ ESBL, BioMérieux, Marcy l’Etoile, France) and CPE selective media (CHROMID^®^ CARBA SMART, BioMérieux), and the plates were incubated aerobically at 37 °C for 24–48 h. Putative *E. coli* colonies of pink to burgundy coloration were sub-cultured on MacConkey agar. Identification and antimicrobial susceptibility testing of the isolates were performed using the Vitek-2 system (BioMérieux), according to the manufacturer’s instructions. The AST-GN96 card was used in order to determine the minimum inhibitory concentration (MIC) of the following antimicrobials: ampicillin, amoxicillin/clavulanic acid, ticarcillin/clavulanic acid, cefalexin, cefalotin, cefoperazone, ceftiofur, cefquinome, imipenem, gentamicin, neomycin, flumequine, enrofloxacin, marbofloxacin, tetracycline, florfenicol, polymyxin B and trimethoprim/sulfamethoxazole. Results were automatically interpreted using the Vitek-2 software (BioMérieux, system version 8.02). Isolates were considered MDR when they exhibited diminished susceptibility to at least one agent of more than three antimicrobial classes.

### 2.3. Phenotypic Confirmation of ESBL or Carbapenemase Production

*E. coli* isolates that presented resistance to third generation cephalosporins (cefoperazone, ceftiofur) were subjected to the double disk synergy test (DDST) for the phenotypic confirmation of ESBL production, according to EUCAST guidelines [26]. A positive result was indicated when the inhibition zones around any of the cephalosporin disks were augmented or when a “keyhole” was formed in the direction of the disk containing clavulanic acid.

Microorganisms that were resistant to imipenem were assessed for phenotypic carbapenemase production using MIC test strips containing meropenem plus phenylboronic acid and meropenem plus ethylenediaminetetraacetic acid (EDTA) (Liofilchem). Isolates that had a ratio meropenem/meropenem plus phenylboronic acid ≥8 or meropenem/meropenem plus EDTA ≥8 were considered positive for class A or class B carbapenemases, respectively.

### 2.4. Antimicrobial Resistance Genotyping of the ESBL- and Carbapenemase-Producing E. coli

Isolates that were found positive in the DDST or the phenotypic carbapenemase tests were characterized using the DNA microarray-based assay CarbaResist from InterArray (FZMB GmbH, Bad Langensalza, Germany). Primer and probe sequences have previously been described in detail [27]. In addition, probes for the detection of the colistin resistance gene family *mcr* were included on the present microarray (see Supplementary File S1). The microarray layout is presented in Supplementary File S2. Protocols and procedures were conducted in accordance with the manufacturer’s instructions (https://www.inter-array.com/Further-Genotyping-Kits, accessed on 10 May 2022). In brief, bacteria were grown overnight on Columbia blood agar, harvested and enzymatically lysed prior to DNA preparation. Genomic DNA from the bacteria was extracted using the Qiagen blood and tissue kit (Qiagen, Hilden, Germany), following the manufacturer’s instructions. The DNA was used in a multiplexed primer elongation, incorporating biotin-16-dUTP. Amplicons were stringently hybridized to the microarray, washed and incubated with a horseradish-peroxidase-streptavidin conjugate. Hybridizations were detected by adding a precipitating dye. An image of the microarray was taken for further analysis.

## 3. Results

### 3.1. Occurrence and Characteristics of ESBL-Producing E. coli

A total of 12 *E. coli* was found to be resistant to third-generation cephalosporins and had a positive ESBL-confirmation test (DDST). The isolates were retrieved from 12 of the 362 sampled wild and feral birds (3.3%) and, in particular, from four Eurasian magpies (*Pica pica*), two Common buzzards (*Buteo buteo*), one Short-toed snake eagle (*Circaetus gallicus*), one Eurasian sparrowhawk (*Accipiter nisus*), one Steppe eagle (*Aquila nipalensis*), one Grey heron (*Ardea cinerea*), one Eurasian Scops owl (*Otus scops*) and one Common swift (*Apus apus*). These birds originated from three regional units of Greece; Korinthia and Messinia that are located in the Peloponnese region of southern Greece, and Magnesia that is located in central Greece.

The 12 ESBL isolates presented resistance to ampicillin and first- to fourth-generation cephalosporins. One exhibited an intermediate phenotype to ticarcillin/clavulanic acid, while none were resistant to carbapenems. Molecular detection of ESBL genes showed that 11 isolates carried the *bla*_CTX-M-1_ group gene alone (*n* = 4) or in combination with *bla*_TEM_ (*n* = 7) and one isolate carried the *bla*_TEM_ alone. The AmpC gene *bla*_ACT_ was detected in four of the isolates and the broad spectrum β-lactamase *bla*_OXA-1_ in only one.

All the isolates were further categorized as MDR. Reduced susceptibility to aminoglycosides was detected in one of the ESBL *E. coli* (1/12, 8.3%) which presented resistance to gentamicin, was intermediate to neomycin and harbored *aphA*. However, the remaining 11 strains were also found to possess aminoglycoside resistance genes and, in particular, *aadA2* (*n* = 7), *aadA4* (*n* = 3) and *rmtA* (*n* = 5).

Eleven of the isolates were additionally non-susceptible to fluoroquinolones (11/12, 91.7%), being either resistant or intermediate to both flumequine and enrofloxacin, while four of them were further resistant to marbofloxacin. The plasmid-mediated quinolone resistance gene *qnrS* was detected in all the fluoroquinolone-resistant *E. coli*.

Trimethoprim/sulfamethoxazole (11/12, 91.7%) resistance was detected in 11 isolates, which harbored a combination of *dfrA* and *sul* variants. In particular, *dfrA5* (*n* = 10) and *sul2* (*n* = 11) were the most common alleles detected, followed by *dfrA17* (*n* = 4) and *sul1* (*n* = 4), respectively.

All 12 isolates were resistant to tetracycline (12/12, 100%), whereas 11 of them harbored the macrolide resistance gene *mrx* alone (*n* = 2) or in combination with *mph* (*n* = 9).

Finally, the *intI1* gene was detected in two strains.

**Figure 1:**
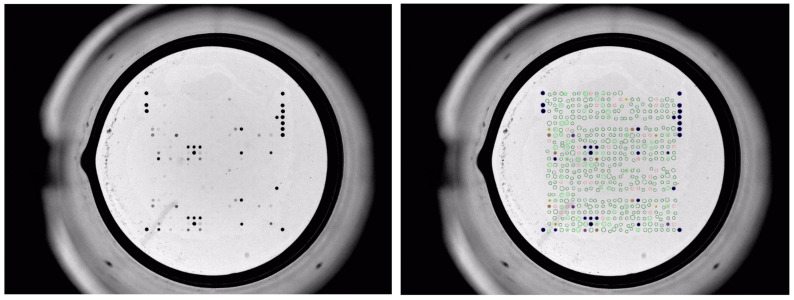
Array results for the ESBL *E. coli* strain obtained from a Common buzzard (**left**), same array with an interpretation grid for fully automated spot recognition (**right**).

The antimicrobial resistance phenotypes and genotypes of the ESBL-producing *E. coli* isolates are summarized in Table 2 and Table 3. An example of the microarray results is depicted in Figure 1.

### 3.2. Occurrence and Characteristics of Carbapenemase-Producing E. coli

Among the 362 samples tested, only one, originating from a Caspian gull (*Larus cachinnans*), yielded a positive culture on the CPE selective media (1/362, 0.3%). The isolated *E. coli* strain was resistant to imipenem and was phenotypically detected to produce MBL in the meropenem-EDTA test.

This isolate was MDR, also being resistant to the other tested β-lactams, as well as to gentamicin, flumequine, enrofloxacin, marbofloxacin and trimethoprim/sulfamethoxazole, while it was susceptible to neomycin and tetracycline.

AMR genotyping revealed that the isolate harbored the carbapenemase gene *bla*_NDM_, as well as genes encoding for other β-lactamases (*bla*_CTX-M-1/15_, *bla*_TEM_, *bla*_OXA-10_). Additionally, genes associated with aminoglycoside (*aadB, aadA1, aadA2, ant2Ia*), quinolone (*qnrB, qnrS*), sulfonamide (*sul1, sul2*), trimethoprim (*dfrA5, dfrA12*) and macrolide (*mph, mrx*) resistance were detected. The isolate also carried the *intI1* gene.

The antimicrobial resistance phenotype and genotype of the carbapenemase-producing *E. coli* is presented in Table 2 and Table 3.

## 4. Discussion

The present study describes the occurrence and the molecular characteristics of ESBL- and carbapenemase-producing *E. coli* recovered from wild and feral birds’ feces in Greece. The incidence of ESBL-producing *E. coli* was rather low (3.3%), which is in accordance with reports from Italy (4/103, 3.9%) [28], Sweden (3/100, 3%) [29], Brazil (5/204, 2.4%) [30] and Alaska (3/76, 3.9%) [31]. The presence of carbapenemase-producing *E. coli* was even lower (0.3%) and comparable to that identified in recent research from other countries, including Spain [32].

However, our results differ significantly from former studies that have reported higher ESBL detection rates in Chile (67/124, 54%) [33], Spain (68/132, 51.5%) [34], Pakistan (26/150, 17.3%) [35] and The Netherlands (51/414, 12.3%) [36], as well as comparatively elevated CPE detection rates in Tunisia (2/150, 1.3%) [12], Algeria (3/32, 9.4%) [37], France (22/158, 13.9%) [38] and Australia (120/504, 23.8%) [39]. This discrepancy could be attributed either to a lower prevalence of such resistant bacteria in the region or to the fact that many prior studies have only been focused on “target” wild bird species, i.e., migratory, omnivorous and aquatic birds. Nevertheless, the systematic review conducted by Chung et al. (2018) has highlighted that, based on the birds migratory status and habitat type, no significant differences in antibiotic resistance rates are observed [40]. Subsequently, in this study, we evaluated ESBL and CPE carriage in several species, including not only high-risk ones, for screening purposes.

ESBL-producing *E. coli* was retrieved from eight different wild bird species. Four species; Common buzzard, Short-toed snake eagle, Steppe eagle and Eurasian sparrow hawk, are raptors and two; Steppe eagle and Eurasian magpie, can be considered omnivores and scavengers. Their diet, which is commonly in direct contact with anthropogenic environments and agricultural animals and waste, constitutes a major potential route for ESBL acquisition [41]. Another alternative pathway is the environment inhabited by the aforementioned species, as also applies to the Grey heron, a waterbird. Aquatic associated species have been documented to be important ESBL hosts as a result of human activities in their habitat [36,42]. Of note, ESBL strains were also detected in samples from a Eurasian Scops owl and a Common swift, two insectivorous birds that live in urbanized areas in vicinity to humans, possibly underlining the importance of human-derived environmental contamination in the dissemination of AMR. Nevertheless, a potential transmission via insects, such as houseflies, cannot be ruled out either [43,44]. Four of the birds that were found to be colonized by ESBL *E. coli* (Short-toed snake eagle, Steppe eagle, Grey heron, Common swift) are also migratory and could thus act as dispersion vectors across long distances [45].

Molecular characterization of the ESBL isolates revealed a dominance of *bla*_CTX-M-1_ group, which is consistent with the majority of published studies on wild avian species to date [12,32,46,47,48,49,50,51]. *E. coli* producing CTX-M group 1 β-lactamases, especially CTX-M-1 and CTX-M-15, has been described as an alarming cause of human infections globally, while variant CTX-M-1 further prevails among food-producing animals [52,53,54]. Notably, we also detected the acquired AmpC gene *bla*_ACT_ in four of the isolates, but not *bla*_CMY-2_, even though prior studies worldwide as well as in Greece have described the latter as the most dominant plasmid encoded AmpC beta-lactamase gene in *Enterobacteriaceae* of wild bird origin [55,56,57]. Co-occurrence of *bla*_ACT_ and *bla*_CTX-M-1_ group has previously been reported in an *Escherichia fergusonii* isolated from a Cattle egret in Tunisia [12].

Of special interest is the fact that a carbapenemase-producing, *bla*_NDM_ positive *E. coli* was identified in the fecal sample of a Caspian gull. The species is one of the most numerous in Greece with a wide range of different feeding habitats. This particular bird was sampled in Porto Lagos, a coastal area in eastern Macedonia, within a wide wetland complex that includes a lagoon, lakes and numerous islands. This site is part of the Ramsar Convention and is regarded as a location of considerable ecological value for breeding and wintering waterbirds and raptors [58]. In Porto Lagos, Caspian gull individuals are either resident or may originate from several other regions, such as the Black Sea, having undertaken a lengthy migration, especially in their first calendar year [59]. Noteworthily, another NDM producing strain, namely a NDM-1 *Klebsiella pneumoniae* recovered from the feces of a Caspian gull, has recently been reported from the Azov-Black Sea in Ukraine [60]. Therefore, we could hypothesize that this resistance gene has been imported to Greece via migratory birds or alternatively that it circulates in the region due to human contamination. In most parts of the world, NDM-type MBLs are sporadically described among humans [3], however, studies in Greece during the past three years have outlined a wide dissemination and establishment of NDM-producing Enterobacteriaceae [61,62]. Given the fact that Caspian gulls frequently feed on anthropogenic food sources such as trawler discards and refuse dumps or nearby livestock-associated facilities such as slaughterhouses, uptake of the resistant strain or the resistance determinants during feeding could also be possible [63]. NDM-producing *E. coli* has also been recovered from other gull species and, in particular, from Yellow-legged gulls (*Larus michahellis*) in Spain, Lesser black-backed gulls (*Larus fuscus*) in Spain and Franklin’s gulls (*Larus pipixcan*) in Chile [60]. Additionally, Black kites (*Milvus migrans*) have been found to harbor *bla*_NDM-5_ *E. coli* in Pakistan and *bla*_NDM-1_ *Salmonella enterica* subsp. *enterica* serovar Corvallis in Germany, the latter being the first ever description of a carbapenemase producer isolated from wild animals [60,64]. Here we report, for the first time in Greece, the detection of a carbapenemase-producing organism from a wild bird, which was identified as NDM positive *E. coli*.

Although our study focused on β-lactamase mediated resistance (ESBLs and carbapenemases), we additionally identified high rates of non-wild-type susceptibility to almost all the antibiotic classes tested, including tetracyclines, sulfonamides, trimethoprim and quinolones. Microarray analysis confirmed the presence of resistance genes against several antibiotics, with variants *sul2*, *dfrA5*, *qnrS*, *mrx* and *aadA2* being the most frequently detected resistance determinants for sulfonamides, trimethoprim, quinolones, macrolides and aminoglycosides, respectively. The detection of multiple AMR genes in all the isolates probably indicates the wide dissemination of AMR determinants in the environment and could be attributed to the presence and cohabitation of transferable plasmids [17,65]. Moreover, two ESBL- and the carbapenemase-producing isolates carried *intI1*, a gene encoding an element known to play a crucial role in the recruitment, spread and expression of resistance genes [66]. Numerous earlier studies have shown an association of both *bla*_CTX-M_ and *bla*_NDM_ with the variable region of class 1 integrons in *E. coli* isolates of human and animal origin [67,68,69,70]. Among wild animals, *intl1* presence is related to their close contact with humans, farm animals and pets [71]. However, previous research has presented a higher incidence of *intl1* among ESBL producers isolated from wildlife varying between 72.4% and 100% [46,50,72], a discrepancy that requires further investigation.

## 5. Conclusions

This study provides evidence of wild birds’ colonization with ESBL- and carbapenemase-producing *E. coli* in Greece. The circulation of *bla*_CTX-M-1_ group, *bla*_TEM_ and *bla*_NDM,_ along with genes conferring resistance to five classes of non β-lactam antimicrobials (fluoroquinolones, trimethoprim, sulfonamides, aminoglycosides, macrolides), poses a serious threat for the spread of MDR bacteria. Thus, wild birds should be regarded as a reservoir, vehicle and indicator of AMR in the environment. Considering that ESBL-PE and CPE detection rates among humans in Greece remain high, regular surveillance studies are required to fully unveil the extent of wild birds’ role in the circulation of such pathogens.

## Figures and Tables

**Table 1:** Number of samples per wild or feral bird species collected and tested.

Family	Common Name	Scientific Name	Number of Samples
Accipitridae	Common buzzard	*Buteo buteo*	4
	Eurasian sparrowhawk	*Accipiter nisus*	1
	Short-toed snake eagle	*Circaetus gallicus*	2
	Steppe eagle	*Aquila nipalensis*	1
Anatidae	Domestic Muscovy duck	*Cairina moschata domestica*	1
	Greater white-fronted goose	*Anser albifrons*	33
	Mallard	*Anas platyrhynchos*	10
	Mute swan	*Cygnus olor*	5
Apodidae	Common swift	*Apus apus*	1
Ardeidae	Grey heron	*Ardea cinerea*	14
	Little egret	*Egretta garzetta*	8
Charadriidae	European golden plover	*Pluvialis apricaria*	1
Columbidae	Domestic pigeon	*Columba livia domestica*	40
	Eurasian collared dove	*Streptopelia decaocto*	18
Corvidae	Hooded crow	*Corvus corone cornix*	5
	Rook	*Corvus frugilegus*	4
	Western jackdaw	*Corvus monedula*	3
	Eurasian magpie	*Pica pica*	79
Emberizidae	Rock bunting	*Emberiza cia*	1
Falconidae	Common kestrel	*Falco tinnunculus*	2
Fringillidae	Common chaffinch	*Fringilla coelebs*	6
	European goldfinch	*Carduelis carduelis*	1
Hirundinidae	Barn swallow	*Hirundo rustica*	1
Laridae	Caspian gull	*Larus cachinnans*	20
	European herring gull	*Larus argentatus*	9
	Yellow-legged gull	*Larus michahellis*	8
	Mediterranean gull	*Larus melanocephalus*	2
	Sandwich tern	*Sterna sandvicensis*	1
Paridae	Great tit	*Parus major*	6
Passeridae	Eurasian tree sparrow	*Passer montanus*	9
	House sparrow	*Passer domesticus*	28
Phalacrocoracidae	Great cormorant	*Phalacrocorax carbo*	4
Phasianidae	Green peafowl	*Pavo muticus*	2
Phoenicopteridae	Greater flamingo	*Phoenicopterus roseus*	5
Phylloscopidae	Leaf warbler	*Phylloscopus* spp.	3
Rallidae	Common moorhen	*Gallinula chloropus*	2
Recurvirostridae	Black-winged stilt	*Himantopus himantopus*	2
Strigidae	Eurasian Scops owl	*Otus scops*	1
	Little owl	*Athene noctua*	2
	Long-eared owl	*Asio otus*	3
Sturnidae	Common starling	*Sturnus vulgaris*	1
Sylviidae	Common whitethroat	*Sylvia communis*	3
	Sardinian warbler	*Sylvia melanocephala*	1
Turdidae	Common blackbird	*Turdus merula*	7
	Redwing	*Turdus iliacus*	1
Tytonidae	Barn owl	*Tyto alba*	1
**Total**			**362**

**Table 2:** Characteristics of the ESBL- and the Carbapenemase-Producing *E. coli*.

Wild Bird Species	Regional Unit of Origin	Antimicrobial Resistance Profile											
		Phenotype		Genotype									
				ESBL Genes	Carbapenemase Genes	AmpC Genes	Other β-Lactamases Genes	Aminoglycoside Resistance Genes	PMQR	Sulfonamide Resistance Genes	Trimethoprim Resistance Genes	Macrolide Resistance Genes	Genes Associated with Mobile Genetic Elements
Magpie (*Pica pica*)	Korinthia	ESBL	AMP, CEX, CF, CFP, CEF, CEQ, FLU *, ENR *, TET, SXT	*bla*_CTX-M-1_ group	-	-	-	*aadA4*	*qnrS*	*sul1, sul2*	*dfrA17*	*mph, mrx*	-
Magpie (*Pica pica*)	Korinthia	ESBL	AMP, CEX, CF, CFP, CEF, CEQ, FLU *, ENR *, TET, SXT	*bla*_CTX-M-__1_ group	-	*bla_ACT_*	-	*aadA4, rmtA*	*qnrS*	*sul1, sul2*	*dfrA5, dfrA17*	*mph, mrx*	-
Magpie (*Pica pica*)	Korinthia	ESBL	AMP, CEX, CF, CFP, CEF, CEQ, FLU *, ENR *, TET, SXT	*bla*_CTX-M-__1_ group	-	*bla_ACT_*	-	*aadA4, rmtA*	*qnrS*	*sul1, sul2*	*dfrA5, dfrA17*	*mph, mrx*	-
Magpie (*Pica pica*)	Korinthia	ESBL	AMP, CEX, CF, CFP, CEF, CEQ, FLU, ENR *, TET, SXT	*bla*_CTX-M-__1_ group	-	*bla_ACT_*	-	*aadA4, rmtA*	*qnrS*	*sul1, sul2*	*dfrA5, dfrA17*	*mph, mrx*	-
Common buzzard (*Buteo buteo*)	Messinia	ESBL	AMP, CEX, CF, CFP, CEF, CEQ, FLU, ENR, MRB, TET, SXT	*bla*_CTX-M-1_ group, *bla*_TEM_	-	*bla_ACT_*	*bla*_OXA-1_	*aadA2, rmtA*	*qnrS*	*sul2*	*dfrA5*	*mph, mrx*	*intl1*
Common buzzard (*Buteo buteo*)	Messinia	ESBL	AMP, CEX, CF, CFP, CEF, CEQ, FLU, ENR *, TET, SXT	*bla*_CTX-M-1_ group, *bla*_TEM_	-	-	-	*aadA2*	*qnrS*	*sul2*	*dfrA5*	*mph, mrx*	-
Grey heron (*Ardea* *cinerea*)	Magnesia	ESBL	AMP, TCC *, CEX, CF, CFP, CEF, CEQ, GEN, NEO *, TET	*bla*_CTX-M-1_ group, *bla*_TEM_	-	-	-	*aphA*	-	*sul2*	-	-	-
Short-toed snake eagle (*Circaetus gallicus*)	Messinia	ESBL	AMP, CEX, CF, CFP, CEF, CEQ, FLU, ENR, MRB, TET, SXT	*bla*_CTX-M-1_ group, *bla*_TEM_	-	-	-	*aadA2*	*qnrS*	*sul2*	dfrA5	*mrx*	-
Eurasian Scops owl (*Otus scops*)	Messinia	ESBL	AMP, CEX, CF, CFP, CEF, CEQ, FLU, ENR, MRB, TET, SXT	*bla*_CTX-M-1_ group, *bla*_TEM_	-	-	-	*aadA2*	*qnrS*	*sul2*	dfrA5	*mph, mrx*	*intI1*
Common swift (*Apus apus*)	Messinia	ESBL	AMP, CEX, CF, CFP, CEF, CEQ, FLU, ENR, MRB, TET, SXT	*bla*_CTX-M-1_ group, *bla_TEM_*	-	-	-	*aadA2*	*qnrS*	*sul2*	dfrA5	*mph, mrx*	-
Eurasian sparrowhawk (*Accipiter nisus*)	Messinia	ESBL	AMP, CEX, CF, CFP, CEF, CEQ, FLU, ENR *, TET, SXT	*bla*_TEM_	-	-	-	*aadA2*	*qnrS*	*sul2*	dfrA5	*mrx*	-
Steppe eagle (*Aquila nipalensis*)	Messinia	ESBL	AMP, CEX, CF, CFP, CEF, CEQ, FLU *, ENR *, TET, SXT	*bla*_CTX-M-1_ group, *bla_TEM_*	-	-	-	*aadA2*	*qnrS*	*sul2*	*dfrA5*	*mph, mrx*	-
Caspian gull (*Larus cachinnans*)	Messinia	CPE	AMP, AMC, TCC, CEX, CF, CFP, CEF, CEQ, IMI, GEN *, FLU, ENR, MRB, SXT	*bla*_CTX-M-1_ group, *bla*_TEM_	*bla*_NDM_	-	*bla*_OXA-10_	*aadB, aadA1,* *aadA2, ant2Ia*	*qnrB, qnrS*	*sul1, sul2*	*dfrA5, dfrA12*	*mph, mrx*	*intI1*

AMP—ampicillin; AMC—amoxicillin/clavulanic acid; TCC—ticarcillin/clavulanic acid; CEX—cefalexin; CF—cefalotin; CFP—cefoperazone; CEF—ceftiofur; CEQ—cefquinome; IMI—imipenem; GEN—gentamicin; NEO—neomycin; FLU—flumequine; ENR—enrofloxacin; MRB—marbofloxacin; TET—tetracycline; SXT—trimethoprim/sulfamethoxazole; * intermediate resistance; PMQR—plasmid-mediated quinolone resistance genes; the isolate did not harbor genes of this category.

**Table 3:** Comparison between the microarray-based genotype and the phenotype obtained by VITEK-2 system.

Detected AMR Genotype	AMR Gene Family	Expected AMR Phenotype	Antibiotics Tested	No of Isolates Harboring the Genotype by Microarray	No of Resistant Isolates by VITEK	No of Susceptible Isolates by VITEK	Concordance (%)
*bla*_CTX-M-1_ group	ESBL	Resistant to 4G/3G cephalosporins, other β-lactams	AMP, AMC, TCC, CEX, CF, CFP, CEF, CEQ	4	4	0	100
*bla*_CTX-M-1_ group, *bla*_TEM_	ESBL	Resistant to 4G/3G cephalosporins, other β-lactams	AMP, AMC, TCC, CEX, CF, CFP, CEF, CEQ	8	8	0	100
*bla*_TEM_ (consensus)	ESBL	Resistant to 4G/3G cephalosporins, other β-lactams	AMP, AMC, TCC, CEX, CF, CFP, CEF, CEQ	1	1	0	100
*bla*_NDM_	Carbapenemases	Resistant to carbapenems, 3G/4G cephalosporins, other β-lactams	IMI, AMP, AMC, TCC, CEX, CF, CFP, CEF, CEQ	1	1	0	100
*bla*_ACT_	AmpC	Resistant to 4G/3G cephalosporins, other β-lactams	AMP, AMC, TCC, CEX, CF, CFP, CEF, CEQ	4	4	0	100
*bla*_OXA-1_	NSBL	Resistant to other β-lactams	AMP, AMC, TCC, CEX, CF	1	1	0	100
*bla*_OXA-10_	NSBL	Resistant to other β-lactams	AMP, AMC, TCC, CEX, CF	1	1	0	100
*aadA4*	Aminoglycosides	Resistant to aminoglycosides	GEN, NEO	1	0	1	0
*aadA2*	Aminoglycosides	Resistant to aminoglycosides	GEN, NEO	6	0	6	0
*aadA2, rmtA*	Aminoglycosides	Resistant to aminoglycosides	GEN, NEO	1	0	1	0
*aadA4, rmtA*	Aminoglycosides	Resistant to aminoglycosides	GEN, NEO	3	0	3	0
*aadB, aadA1, aadA2, ant2Ia*	Aminoglycosides	Resistant to aminoglycosides	GEN, NEO	1	1	0	100
*aphA*	Aminoglycosides	Resistant to aminoglycosides	GEN, NEO	1	1	0	100
*qnrS*	Quinolones	Resistant to fluoroquinolones	FLU, ENR, MRB	11	11	0	100
*qnrB, qnrS*	Quinolones	Resistant to fluoroquinolones	FLU, ENR, MRB	1	1	0	100
*sul1, sul2, dfrA17*	Sulfonamides -Trimethoprim	Resistant to sulfonamides/trimethoprim	SXT	1	1	0	100
*sul1, sul2, dfrA5, dfrA12*	Sulfonamides -Trimethoprim	Resistant to sulfonamides/trimethoprim	SXT	1	1	0	100
*sul1, sul2, dfrA5, dfrA17*	Sulfonamides -Trimethoprim	Resistant to sulfonamides/trimethoprim	SXT	3	3	0	100
*sul2, dfrA5*	Sulfonamides -Trimethoprim	Resistant to sulfonamides/trimethoprim	SXT	7	7	0	100
*sul2*	Sulfonamides -Trimethoprim	Susceptible to sulfonamides/trimethoprim	SXT	1	0	1	100
**Overall concordance (mean)**				**80%**			

AMP—ampicillin; AMC—amoxicillin/clavulanic acid; TCC—ticarcillin/clavulanic acid; CEX—cefalexin; CF—cefalotin; CFP—cefoperazone; CEF—ceftiofur; CEQ—cefquinome; IMI—imipenem; GEN—gentamicin; NEO—neomycin; FLU—flumequine; ENR—enrofloxacin; MRB—marbofloxacin; SXT—trimethoprim/sulfamethoxazole; NSBL—narrow spectrum β-lactamases.

## Data Availability

All data generated for this study are presented within the manuscript and the supplementary files.

## Supplementary Materials

The following supporting information can be downloaded at: https://www.mdpi.com/article/10.3390/microorganisms10061217/s1, Supplementary File S1: Genes detected by the CarbaResist DNA microarray-based assay. Supplementary File S2: CarbaResist microarray layout.
