# Supplementary material for: Occurrence and Characteristics of ESBL- and Carbapenemase- Producing Escherichia coli from Wild and Feral Birds in Greece
Source: Microorganisms. 2022 Jun 14;10(6):1217. doi: 10.3390/microorganisms10061217 (PMC9227375; doi:10.3390/microorganisms10061217)
Supplement: Supplementary file 1 [file microorganisms-10-01217-s001.zip › Supplementary File S1. Genes Detected by the CarbaResist DNA microarray-based assay.pdf]

Supplementary File S1: Genes detected by the CarbaResist DNA microarray-based assay.

Target Gene	Gene Function	Accession Number
gad	glutamate decarboxylase of *Escherichia coli*	AE014075.1
ihfA	integration host factor subunit alpha	U00096.3
dnaE	DNA polymerase III subunit alpha	U00096.3
basC	acinetobactin biosynthesis protein of *Acinetobacter baumannii*	AY571146.1
efp	elongation factor P of *Acinetobacter baumannii*	CP001172.1
pld	phospholipase D of *Acinetobacter baumannii*	CP000521.1
cfa	colicin five activity protein of *Citrobacter freundii* and *Citrobacter braakii*	U09771.1
ecfX	extracytoplasmic function sigma factor of *Pseudomonas aeruginosa* [LavenirR-JocktaneD-2007]	DQ996558.1
invA	invasin A, highly specific for genus *Salmonella*	CP000026.1
ipaH9.8	invasion plasmid antigen	AF047365.1
khe	klebsolysin of *Klebsiella pneumoniae*	AF293352.1
lacY	lactose permease; the *lacY* gene is missing in all *Shigella spp*.	U00096.2
rrs	16S rRNA	U00096.3
*bla*BIC	carbapenemase, class A beta-lactamase	GQ260093.1
*bla*DIM	carbapenemase, class B metallo beta-lactamase	KC004136.2
*bla*GES	carbapenemase, class A beta-lactamase	AY219651.1
*bla*GIM	carbapenemase, class B metallo beta-lactamase	consensus
*bla*GOB	carbapenemase, class B metallo beta-lactamase	consensus
*bla*IMI-3 (NmcA)	carbapenemase, class A beta-lactamase associated with imipenem resistance	AY780889.1
*bla*IMI-R	regulator of *bla*IMI-3 (NMC-A)	Z21956.1
*bla*IMP	carbapenemase, class B metallo beta-lactamase	consensus
*bla*IMP25 (*bla*SIM-1)	carbapenemase, class B metallo beta-lactamase (synonym: *bla*SIM)	EU686387.1
*bla*IMP35	carbapenemase, class B metallo beta-lactamase	JQ432564.1
*bla*IND	carbapenemase, class B metallo beta-lactamase of Chryseobacterium	consensus
*bla*KHM	carbapenemase, class B metallo beta-lactamase	consensus
*bla*KPC	carbepenemase, class A serin beta-lactamase	consensus
*bla*NDM	carbapenemase, class B metallo beta-lactamase (New Dehli metallo beta-lactamase)	consensus
*bla*PAM-1	carbapenemase, subclass B3 metallo beta-lactamase (Pseudomonas alcaligenes metallo-betalactamase)	AB858498.1
*bla*SFH-1	carbapenemase, class B metallo beta-lactamase	AF197943.1
*bla*SMB-1	carbapenemase, class B metallo beta-lactamase	AB636283.1
*bla*SME	carbapenemase, class A beta-lactamase	consensus
*bla*SPM-1	carbapenemase, class B metallo beta-lactamase	AY341249.1
*bla*VIM	carbapenemase, class B metallo beta-lactamase	consensus
*bla*VIM-2	carbapenemase, class B metallo beta-lactamase	AF191564.1
*bla*VIM-7	carbapenemase, class B metallo beta-lactamase	AJ536835.1
*bla*OXA-23-like	carbapenemase, class D beta-lactamase	AJ132105.1
*bla*OXA-40-like	carbapenemase, class D beta-lactamase	AF509241.1
*bla*OXA-48-like	carbapenemase, class D beta-lactamase	AY236073.2
*bla*OXA-51-like	carbapenemase, class D beta-lactamase	CP000863.1
ISABa1 to *bla*OXA-51	Insertion sequence ABa1 is adjacent to *bla*OXA-51-like gene. This combination mediated carbapenem resistant in Acinetobacter baumannii isolates	CP001921.1
no ISABa1 to *bla*OXA-51	Insertion sequence ABa1 is not adjacent to *bla*OXA-51-like gene. This combination does not mediate carbapenem resistant in Acinetobacter baumannii isolates	CU459141.1
*bla*OXA-54	carbapenemase, class D beta-lactamase	AY500137.1
*bla*OXA-55	carbapenemase, class D beta-lactamase	AY343493.1
*bla*OXA-58	carbapenemase, class D beta-lactamase	AY665723.1
*bla*OXA-134/235/284	carbapenemase *bla*OXA-134 or -235 or -284, class D beta-lactamase	AYHO01000005.1

**Target Gene**	**Gene Function**	**Accession Number**
*bla*OXA-143/182/253/255	carbapenemase *bla*OXA-40-like, class D beta-lactamase	GQ861437.1
*bla*OXA-181/232	carbapenemase *bla*OXA-48-like, class D beta-lactamase	CP000469.1
*bla*OXA-214	carbapenemase, class D beta-lactamase	JN861783.1
*bla*OXA-279	carbapenemase, class D beta-lactamase	APOK01000044.1
*bla*OXA-292	carbapenemase, class D beta-lactamase	APRH01000012.1
*bla*CME	extended spectrum beta-lactamase, class A	AF033200.1
*bla*CTX-M1/15	extended spectrum beta-lactamase, class A	X92506.1, HQ202266.1
*bla*CTX-M2	extended spectrum beta-lactamase, class A	AF286192.1
*bla*CTX-M8	extended spectrum beta-lactamase, class A	AY750914.2
*bla*CTX-M9	extended spectrum beta-lactamase, class A	FQ482074.1
*bla*MOX-CMY9	extended-spectrum beta-lactamase precursor, class C	AF381617.1
*bla*PER-1	extended-spectrum beta-lactamase, class A beta-lactamase PER-1	Z21957.1
*bla*PER-2	extended-spectrum beta-lactamase, class A beta-lactamase PER-2	X93314.1
*bla*SHV	class A beta-lactamase - consensus sequence for *bla*SHV genes, including extended-spectrum beta-lactamases	consensus
*bla*TEM	class A beta-lactamase - consensus sequence for *bla*TEM genes, including extended-spectrum beta-lactamases	consensus
*bla*VEB	extended-spectrum beta-lactamase, class A	consensus
*bla*OXA-18	extended spectrum beta-lactamase, class D	EU503121.1
*bla*OXA-45	extended spectrum beta-lactamase, class D	AJ519683.1
*bla*OXA-1	narrow spectrum beta-lactamase, class D	AY458016.1
*bla*OXA-9	narrow spectrum beta-lactamase, class D	M55547.1
*bla*OXA-2	consensus probe for extended and narrow spectrum class D beta-lactamases belonging to group *bla*OXA-2	consensus
*bla*OXA-10	consensus probe for extended and narrow spectrum class D beta-lactamases belonging to group *bla*OXA-10	consensus
*bla*OXA-60	narrow spectrum beta-lactamase, class D	AF525303.2
*bla*MIR	extended spectrum beta-lactamase, class C beta-lactamase	M37839.2
*bla*ACC	AmpC beta-lactamase	EF554600.1
*bla*ACT	AmpC beta-lactamase	U58495.2
*bla*CMY	AmpC beta-lactamase, consensus sequence	consensus
*bla*DHA	AmpC beta-lactamase	EF406115.1
*bla*FOX	AmpC beta-lactamase	consensus
*bla*MOX	AmpC beta-lactamase	consensus
aac(3')	3-N-aminoglycoside acetyltransferase; associated with resistance to astromicin; gentamicin; sisomicin	consensus
aac(3')-Ia	3-N-aminoglycoside acetyltransferase; associated with resistance to astromicin; gentamicin; sisomicin	U90945.1
aac(3')-Ib	3-N-aminoglycoside acetyltransferase; associated with resistance to astromicin; gentamicin; sisomicin	KJ679408.1
aac(3')-Ic	3-N-aminoglycoside acetyltransferase; associated with resistance to astromicin; gentamicin; sisomicin	AJ511268.1
aac(3')-Ie	3-N-aminoglycoside acetyltransferase; associated with resistance to astromicin; gentamicin; sisomicin	AY458224.1
aac(3')-IVa	3-N-aminoglycoside acetyltransferase; associated with resistance to apramycin; dibekacin; gentamicin; netilmicin; sisomicin; tobramycin	EU784152.1
aac(6')	aminoglycoside 6'-N-acetyltransferase, associated with resistance to amikacin; dibekacin; isepamicin; netilmicin; sisomicin; tobramycin	consensus
aac(6')-31	aminoglycoside 6'-N-acetyltransferase; associated with resistance to streptomycin, spectinomycin	AJ640197.1
aac(6')-Ib	aminoglycoside 6'-N-acetyltransferase; associated with resistance to streptomycin, spectinomycin	M21682.1
aac(6')-II	aminoglycoside 6'-N-acetyltransferase; associated with resistance to streptomycin, spectinomycin	EF127959.1
aac(6')-IIa	aminoglycoside 6'-N-acetyltransferase; associated with resistance to streptomycin, spectinomycin	EU912537.1
aac(6')-IIc	aminoglycoside 6'-N-acetyltransferase; associated with resistance to streptomycin, spectinomycin	EU855788.1
aac-aph	6'-aminoglycoside-N-acetyltransferase/2''-aminoglycoside phosphotransferase; associated with resistance to gentamycin	AE017171.1
aadA1	aminoglycoside adenyltransferase; associated with resistance to streptomycin, spectinomycin	EU704128.1
aadA2	aminoglycoside adenyltransferase; associated with resistance to streptomycin, spectinomycin	EU704128.1
aadA4	aminoglycoside adenyltransferase; associated with resistance to streptomycin, spectinomycin	Z50802.3
aadB	2''-aminoglycoside nucleotidyltransferase	L06418.4
ant2	aminoglycoside (2'') adenylyltransferase; associated with resistance to dibekacin; gentamicin; kanamycin;	L06418.4

Target Gene	Gene Function	Accession Number
	sisomicin; tobramycin	
aphA	aminoglycoside 3'-phosphotransferase; kanamycin resistance protein	AY260546.3
armA	16S rRNA methylase, associated with aminoglycoside resistance	AB117519.1
grm	16S rRNA methylase, associated with gentamicin resistance	M55521.1
npmA	16S rRNA methylase, associated with aminoglycoside resistance	AB261016.1
rmtA	16S rRNA methylase, associated with aminoglycoside resistance	AB083212.2
rmtB	16S rRNA methylase, associated with aminoglycoside resistance	DQ345788.1
rmtC	16S rRNA methylase, associated with aminoglycoside resistance	AB194779.2
rmtD	16S rRNA methylase, associated with aminoglycoside resistance	DQ914960.2
mph	macrolide 2'-phosphotransferase	consensus
mrx	member of macrolide inactivation gene cluster *mphA – mrx - mphR*	consensus
qepA	QepA - fluoroquinolone/quinolone efflux pump	AM886293.1
qnrA1	quinolone or fluoroquinolone resistance protein	AY931018.1
qnrB	quinolone or fluoroquinolone resistance protein	AB281054.1
qnrC	quinolone or fluoroquinolone resistance protein	EU917444.1
qnrD	quinolone or fluoroquinolone resistance protein	FJ228229.1
qnrS	quinolone or fluoroquinolone resistance protein	AM234722.1
sul1	dihydropteroate synthetase type 1	AJ698325.1
sul2	dihydropteroate synthetase type 2	DQ464881.1
sul3	dihydropteroate synthetase type 3	AJ459418.2
dfrA1	dihydrofolate reductase type 1	AJ884723.1
dfrA5	dihydrofolate reductase type 5	AB188269.1
dfrA7	dihydrofolate reductase type 7	AB161450.1, AM237806.1
dfrA12	dihydrofolate reductase type 12	AB154407.1
dfrA13	dihydrofolate reductase type 13 (synonym A21)	Z50802.3
dfrA14	dihydrofolate reductase type 14	AJ313522.1
dfrA15	dihydrofolate reductase type 15	Z83311.1
dfrA17	dihydrofolate reductase type 17	AF169041.1
dfrA19	dihydrofolate reductase type 19	AJ310778.1
intI1	class 1 integron integrase	AY260546.3
intI2	class 2 integron integrase	AY183453.1
intI3	class 3 integron integrase	EF469602.1
tnpISEcp1	transposase for the transposon ISEcp1	AB543698.1
oqxA	OqxA - membran fusion protein, component of RND-type multidrug efflux pump, associated with olaquindox resistance	EU370913.1
oqxB	OqxB - integral membrane protein, component of RND-type multidrug efflux pump, associated with olaquindox resistance	EU370913.1
higA	higA is the antitoxin of the translation-dependent mRNA interferase toxin higB	U43847.1
higB	Ectopic expression of higB causes inhibition of cell growth which is alleviated by co-expression of higA	U43847.1
splA	splA is the antitoxin of the translation-dependent mRNA interferase toxin splT	EU294228.1
splT	Ectopic expression of splT causes inhibition of cell growth which is alleviated by co-expression of splA	EU294228.1
mcr-1 / mcr-2	mcr-1 and mcr-2, phosphoethanolamine transferase associated with resistance to colistin and polymyxin-type antibiotics	KP347127.1
mcr-3	mcr-3, phosphoethanolamine transferase associated with resistance to colistin and polymyxin-type antibiotics	KY924928.1
mcr-4	mcr-4, phosphoethanolamine transferase associated with resistance to colistin and polymyxin-type antibiotics	MF543359.1.1
mcr-5	mcr-5, phosphoethanolamine transferase associated with resistance to colistin and polymyxin-type antibiotics	NG_055658.1
mcr-6	mcr-6, phosphoethanolamine transferase associated with resistance to colistin and polymyxin-type antibiotics	NG_055781.1
mcr-7	mcr-7, phosphoethanolamine transferase associated with resistance to colistin and polymyxin-type antibiotics	NG_056413.1
mcr-8	mcr-8, phosphoethanolamine transferase associated with resistance to colistin and polymyxin-type antibiotics	NG_061399.1
mcr-9	mcr-9, phosphoethanolamine transferase associated with resistance to colistin and polymyxin-type antibiotics	MK070339.1

Filename: Supplementary File S1. Genes Detected by the CarbaResist DNA microarray-based assay.docx
Directory: E:\6.13\membranes-1741051
Template: C:\Users\MDPI\AppData\Roaming\Microsoft\Templates\Normal.dotm
Title:
Subject:
Author: Sascha Braun
Keywords:
Comments:
Creation Date: 11/17/2016 11:08:00 PM
Change Number: 8
Last Saved On: 5/13/2022 4:10:00 PM
Last Saved By: ATHANASAKOPOULOU ZOI
Total Editing Time: 0 Minutes
Last Printed On: 6/14/2022 4:00:00 PM
As of Last Complete Printing
Number of Pages: 3
Number of Words: 1,769 (approx.)
Number of Characters: 9,716 (approx.)
