# Supplementary figures and images for: Occurrence and Characteristics of ESBL- and Carbapenemase- Producing Escherichia coli from Wild and Feral Birds in Greece
Source: Microorganisms. 2022 Jun 14;10(6):1217. doi: 10.3390/microorganisms10061217 (PMC9227375; doi:10.3390/microorganisms10061217)

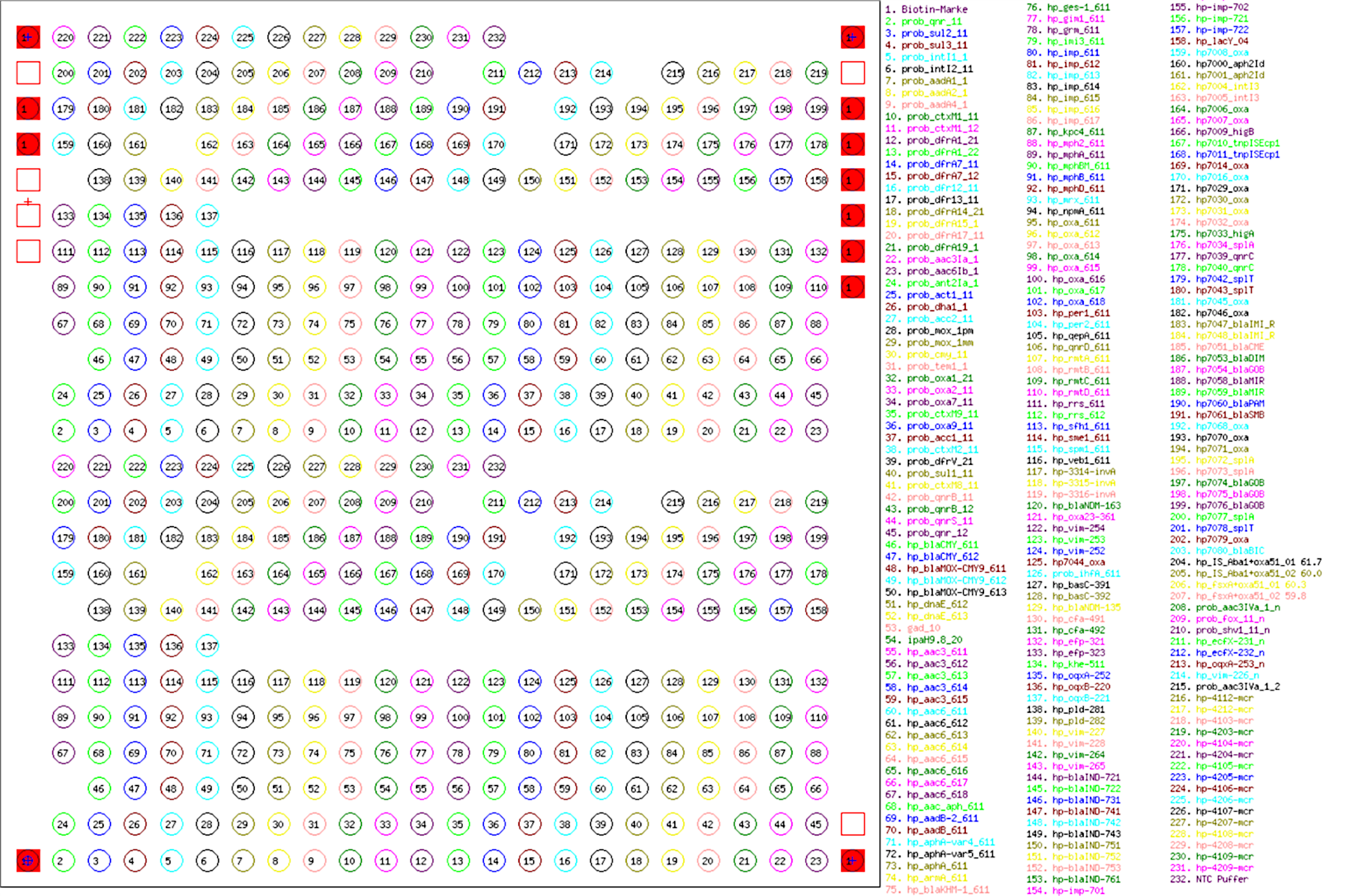

Supplement: Supplementary file 1 [file microorganisms-10-01217-s001.zip › Supplementary File S2. Microarray Layout.tif]
